# Supplementary material for: Mechanisms governing the pioneering and redistribution capabilities of the non-classical pioneer PU.1
Source: Nat Commun. 2020 Jan 21;11:402. doi: 10.1038/s41467-019-13960-2 (PMC6972792; doi:10.1038/s41467-019-13960-2)
Supplement: Supplementary file 7 — Source data [file 41467_2019_13960_MOESM7_ESM.zip › Source_Data/FigureS2/FigureS2B_MotifScanOutput/KmeansCluster5/homerResults/motif22.similar.html]

motif22

## Information for motif22

G
A
T
C
G
C
T
A
T
C
G
A
G
A
T
C
A
G
T
C
C
G
T
A
A
G
T
C
T
G
C
A
G
C
A
T
  
Reverse Opposite:  

C
G
T
A
A
C
G
T
T
C
A
G
A
C
G
T
C
T
A
G
C
T
A
G
A
G
C
T
C
G
A
T
C
T
A
G
  

|  |  |
| --- | --- |
| p-value: | 1e-46 |
| log p-value: | -1.065e+02 |
| Information Content per bp: | 1.677 |
| Number of Target Sequences with motif | 926.0 |
| Percentage of Target Sequences with motif | 23.27% |
| Number of Background Sequences with motif | 6110.6 |
| Percentage of Background Sequences with motif | 14.27% |
| Average Position of motif in Targets | 98.8 +/- 56.6bp |
| Average Position of motif in Background | 100.3 +/- 67.7bp |
| Strand Bias (log2 ratio + to - strand density) | -0.0 |
| Multiplicity (# of sites on avg that occur together) | 1.11 |
| Motif File: | file (matrix) reverse opposite |

### Similar de novo motifs found

|  |  |  |  |  |  |  |  |
| --- | --- | --- | --- | --- | --- | --- | --- |
| Rank | Match Score | Redundant Motif | P-value | log P-value | % of Targets | % of Background | Motif file |
| 1 | 0.771 | C G A T C T G A A G T C A G T C C T G A A G T C T C A G | 1e-30 | -69.325142 | 47.41% | 38.03% | motif file (matrix) |
| 2 | 0.903 | G A T C C T G A C G T A A G T C A G T C C G T A A G T C C T A G | 1e-12 | -28.972792 | 5.48% | 3.15% | motif file (matrix) |
